# Enhanced mitochondrial DNA editing in mice using nuclear-exported TALE-linked deaminases and nucleases

**DOI:** 10.1186/s13059-022-02782-z

**Published:** 2022-10-12

**Authors:** Seonghyun Lee, Hyunji Lee, Gayoung Baek, Eunji Namgung, Joo Min Park, Sanghun Kim, Seongho Hong, Jin-Soo Kim

**Affiliations:** 1 Center for Genome Engineering, Institute for Basic Science, Daejeon, 34126 Republic of Korea; 2 Laboratory Animal Resource and Research Center, Korea Research Institute of Bioscience and Biotechnology, Cheongju, 28116 Republic of Korea; 3 School of Medicine, Sungkyunkwan University, Suwon, 16419 Republic of Korea; 4 Center for Cognition and Sociality, Institute for Basic Science, Daejeon, 34126 Republic of Korea; 5 College of Veterinary Medicine and Research Institute of Veterinary Medicine, Chungbuk National University, Cheongju, 28644 Republic of Korea

**Keywords:** DdCBE, NES, mitoTALEN, mtDNA, Mitochondrial DNA editing

## Abstract

We present two methods for enhancing the efficiency of mitochondrial DNA (mtDNA) editing in mice with DddA-derived cytosine base editors (DdCBEs). First, we fused DdCBEs to a nuclear export signal (DdCBE-NES) to avoid off-target C-to-T conversions in the nuclear genome and improve editing efficiency in mtDNA. Second, mtDNA-targeted TALENs (mitoTALENs) are co-injected into mouse embryos to cleave unedited mtDNA. We generated a mouse model with the m.G12918A mutation in the MT-ND5 gene, associated with mitochondrial genetic disorders in humans. The mutant mice show hunched appearances, damaged mitochondria in kidney and brown adipose tissues, and hippocampal atrophy, resulting in premature death.

**Supplementary Information:**

The online version contains supplementary material available at 10.1186/s13059-022-02782-z.

## Background

Mitochondrial DNA (mtDNA), an extrachromosomal genome in an organelle, encodes 13 polypeptides for the oxidative phosphorylation system, 22 tRNAs, and 2 ribosomal RNAs in a 16.5-kb circular double-stranded DNA in both humans and mice [1, 2]. Various genetic diseases caused by human mtDNA mutations have been reported, which include mitochondrial encephalopathy, lactic acidosis and stroke-like episodes (MELAS), Leber hereditary optic neuropathy (LHON), and Leigh syndrome, collectively affecting one in 5000 individuals. Most of these mitochondrial diseases are associated with severe symptoms, including encephalopathy, myopathy, hearing loss, or vision loss [3]. Furthermore, mtDNA mutations are often considered a significant cause of diabetes and aging [4, 5].

We and others recently reported mitochondrial genome editing in human cells [6, 7], animals [8–12], and plants [13–15] using DddA_tox_-derived cytosine base editors (DdCBEs), which enable targeted C-to-T conversions and are composed of the split interbacterial toxin DddA_tox_, custom-designed DNA-binding transcription activator-like effector (TALE) arrays [16], a uracil glycosylase inhibitor (UGI), and a mitochondrial targeting sequence (MTS). In this study, we sought to improve mtDNA editing efficiency in mouse embryos and to avoid unwanted off-target editing in the nuclear genome.

## Results and discussion

**Fig. 1:**
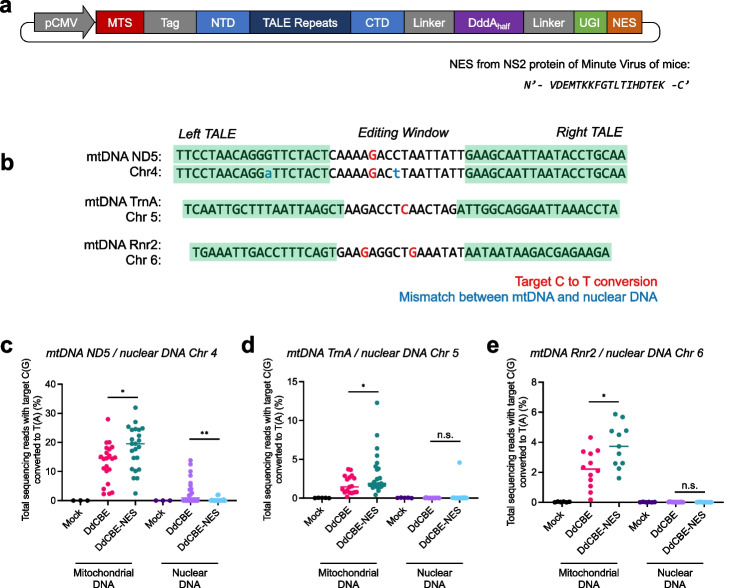
Improved cytosine base editing efficiency in mouse blastocysts. **a** Plasmid construct expressing DdCBE-NES. The amino acid sequence of the NES from the MVM NS2 protein is shown. **b** Base editing targets in the mitochondrial and nuclear genomes. C(G)-to-T(A) conversion targets are shown in red, and mismatches between mtDNA and nuclear DNA are shown in lowercase blue font. **c**–**e** Target C(G)-to-T(A) editing efficiencies in mouse blastocysts. The sequencing data were obtained from cultured blastocysts that developed after one-cell stage embryos were microinjected with mRNA encoding the DdCBE or DdCBE-NES. mtDNA and nuclear DNA amplicons were respectively analyzed for the *MT-ND5* gene and a potential off-target site on chromosome 4 (**c**), the *MT-TrnA* gene and an identical target site on chromosome 5, and *MT-Rnr2* and an identical target site on chromosome 6. For mitochondrial-specific amplicons, data points from DdCBE-injected blastocysts are shown as magenta and those from DdCBE-NES-injected blastocysts as green. For nuclear DNA-specific amplicons, purple indicates DdCBE and light blue DdCBE-NES. All data sets represented in the graphs were obtained from at least three biologically independent samples. The exact *p*-values are *0.0189 and **0.0045 for **c**, *0.0327 for **d**, and *0.0881 for **e**. (*N* ≥ 3; n.s., not significant, *p < 0.05, **p < 0.01, and ***p < 0.001 using Student’s two-tailed *t*-test)

We reasoned that the addition of a nuclear export signal (NES) to DdCBEs could enhance base editing efficiency in the mitochondria and reduce off-target editing in the nuclear genome. To this end, we attached the NES sequence, N′-VDEMTKKFGTLTIHDTEK-C′, derived from the NS2 protein of minute virus of mice (MVM NES) [17], to the C-terminus of DdCBEs to facilitate protein export out of the nuclear membrane (Fig. 1a). We first chose the DdCBE targeted to the *MT-ND5* gene to induce the m.G12918A mutation, associated with mitochondrial genetic disorders. This DdCBE may also recognize an off-target site with a single-nucleotide mismatch in the nuclear genome (chromosome 4: 80,002,764–80,002,825, GRCm38.p6 assembly) (Fig. 1b, top). We microinjected mRNA encoding DdCBE containing MTS or DdCBE containing NES in addition to MTS (termed DdCBE-NES, hereinafter) into mouse zygotes and cultured the zygotes to the blastocyst stage. We then measured C-to-T editing efficiencies at the target site in mtDNA and the potential off-target site in nuclear DNA in each blastocyst. As shown in Fig. 1c, the addition of NES to DdCBE improved mtDNA editing efficiency by 38.9% (= 18.2%/13.1%). Thus, the DdCBE-NES achieved an editing frequency of 18.2% in a total of 23 blastocysts, whereas the DdCBE showed an editing frequency of 13.1% in a total of 22 blastocysts. Importantly, the NES attachment to DdCBE reduced off-target C-to-T conversions in the nuclear genome. Thus, the DdCBE induced off-target edits in nuclear DNA in 11 out of 22 blastocysts with frequencies of up to 13.8% (6.4 ± 1.3%, on average). In contrast, the DdCBE-NES induced off-target edits in only two out of 23 blastocysts with low frequencies of 2.0% and 1.9% (2.0 ± 0.1%, on average) (Additional file 1: Table S1). Furthermore, we conducted whole-genome sequencing for mice injected with DdCBE or DdCBE-NES. A recent study revealed that the use of NES largely avoided TALE-dependent and TALE-independent off-target editing in human cells [18]. Therefore, we chose only DdCBE-mediated mutations in nuclear DNA, 5′-TC(GA)-3′ to 5′-TT(AA)-3′, then filtered out homozygous mutations and read depths below 20×. The off-target read ratio in the nuclear genome was 0.018% for the wild-type C57BL/6J mouse. However, DdCBE-treated mice showed elevated levels of off-target mutations, such that the off-target ratios were 0.027% and 0.020% for Pt120 and Pt121, respectively (Additional file 1: Fig. S1). On the other hand, mice microinjected with mRNA encoding DdCBE-NES (NES-Pt) demonstrated comparable off-target ratios with the wild-type, such that the ratios were 0.018% and 0.017% for NES-Pt-1 and NES-Pt-2, respectively. Note that NES-Pt mice showed higher on-target m.G12918A editing efficiencies than mice treated with DdCBE lacking NES, but the off-target ratios were on par with those in the wild type.

We also compared DdCBE-NES with DdCBE targeted to two other genes in mouse embryos: *MT-TrnA* and *MT-Rnr2*. As shown in Fig. 1d and e, DdCBE-NES was more efficient than DdCBE. Thus, the *TrnA*-targeted DdCBE and DdCBE-NES achieved base editing with an average frequency of 1.8 ± 0.3% and 3.2 ± 0.6%, respectively, whereas the *Rnr2*-targeted DdCBE and DdCBE-NES achieved base editing with an average frequency of 2.2 ± 0.4% and 3.8 ± 0.4%, respectively. Neither DdCBE-NES nor DdCBE induced off-target C-to-T conversions at a nuclear site, whose DNA sequence is 100% identical with the mtDNA target sequence. Note that both DdCBE-NES and DdCBE contain the MTS derived from SOD2 and COX8A, and lack a nuclear localization signal (NLS), which prevents unwanted off-target editing in the nuclear genome, albeit not always. Our results suggest that it is precautionary to use DdCBE-NES rather than DdCBE to avoid unwanted editing in the nuclear genome.

**Fig. 2:**
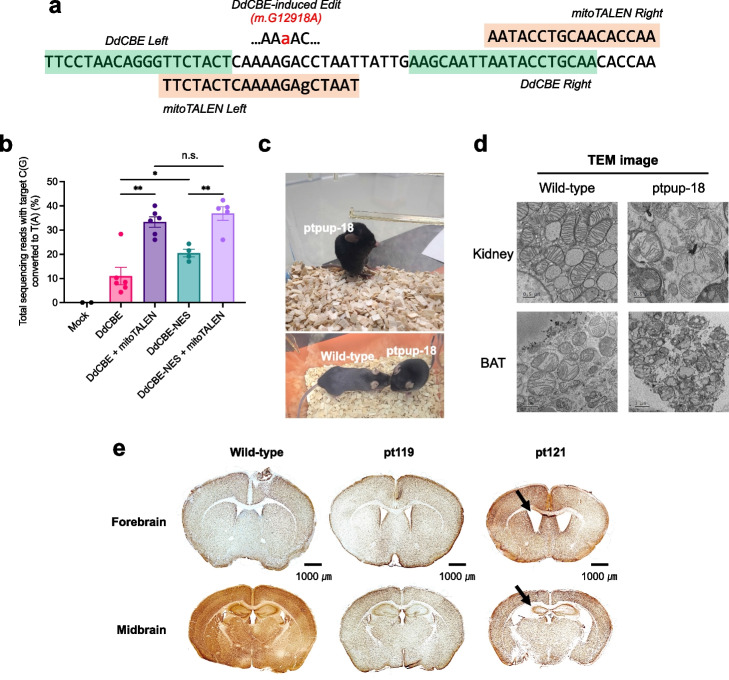
Improved editing efficiency of DdCBE-NES in the presence of mitoTALEN in mice. **a** Base editing target for generating the m.G12918A mutation. The TALE binding sequences for the DdCBE are highlighted in green and for the mitoTALEN in orange. The mitoTALEN was designed to recognize a site with a single mismatch with the wild-type mtDNA sequence, as denoted with a lowercase letter. **b** Effect of mitoTALEN, used to eliminate wild-type mtDNA in mouse blastocysts, on the m.G12918A base editing efficiency. Targeted deep sequencing data were obtained from blastocysts that developed after microinjection of zygotes with mRNAs encoding the indicated constructs. Exact *p*-values are **0.0006 for DdCBE compared with DdCBE + mitoTALEN, **0.0022 for DdCBE-NES compared with DdCBE-NES + mitoTALEN, and *0.0467 for DdCBE compared with DdCBE-NES; n.s. is 0.3519. (*N* ≥ 3; n.s., not significant, *p < 0.05, **p < 0.01, and ***p < 0.001 using Student’s two-tailed *t*-test). **c** Images of a mouse harboring the m.G12918A point mutation (ptpup-18) with a wild-type C57BL/6 mouse. **d** Transmission electron micrographs of the mitochondria in the kidney and brown adipose tissue from wild-type and ptpup-18 mice. **e** Immunohistochemistry images of the brain sections from wild-type and m.G12918A mutant mice. Brain sections, stained with anti-NeuN antibody visualized with DAB (3,3′-diaminobenzidine), from the forebrain and midbrain regions of brains from a wild-type mouse, pt119 (a littermate of the pt121 mouse with normal behavior), and the pt121 mouse with a hunchback phenotype. The arrow on the pt121 forebrain section indicates one of the enlarged lateral ventricles, and the arrow on the pt121 midbrain section shows the asymmetric hippocampus in this mouse harboring the m.G12918A mutation

Mitochondrial genome-targeted TALE nucleases (mitoTALENs) and zinc finger nucleases have been successfully used to cleave mutant mtDNA selectively and, thereby, reduce mutant mtDNA loads in a heteroplasmic state. We reasoned that mitoTALENs designed to cleave the wild-type sequence, conversely, could increase the frequency of mtDNA edits induced by DdCBEs. To make sure that mitoTALEN cannot cleave mutant mtDNA containing the m.G12918A mutation induced by DdCBE or DdCBE-NES and that it still cleaves wild-type mtDNA, we intentionally constructed our mitoTALEN to recognize a mismatched sequence. Thus, the resulting mitoTALEN was designed to recognize a sequence that contains a single-nucleotide mismatch with the wild-type sequence and two nucleotide mismatches with the m.G12918A mutant sequence. We microinjected mRNA encoding DdCBE or DdCBE-NES alone or together with mRNA encoding the mitoTALEN into one-cell stage mouse embryos and measured mtDNA editing frequencies in blastocysts via high-throughput sequencing. Co-injection of the mitoTALEN enhanced editing frequencies of DdCBE or DdCBE-NES significantly. Thus, the m.G12918 mutation was obtained with frequencies of 11.0 ± 3.6% (DdCBE) or 20.5 ± 1.6% (DdCBE-NES) without mitoTALEN, whereas the mutation was obtained with frequencies of 33.3 ± 2.1% (DdCBE) or 36.8 ± 2.8% (DdCBE-NES) with mitoTALEN (Fig. 2b). Due to adjacent DNA-binding sites, it is possible that mitoTALENs interfere with DdCBEs. Nevertheless, our results showed that simultaneous use of DdCBEs and mitoTALENs could enhance the mutation frequency in mouse embryos.

In humans, the m.G13513A mutation, corresponding to the m.G12918A mutation in murine mtDNA, is associated with Leigh disease, MELAS syndrome, and LHON-MELAS overlap syndrome [19, 20]. The m.G13513A mutation (and m.G12918A in murine mtDNA) causes a D393N amino acid change in the mtND5 protein. To investigate whether the mtDNA mutation in mice causes any phenotypic changes, we implanted base-edited embryos into surrogate mothers and obtained a total of 12 F_0_ offspring with the m.G12918A mutation (Additional file 1: Table S2). Mutation frequencies ranged from 4.1 to 46.2% in these mice. Most of these mutant mice appeared normal and did not display any noticeable phenotypes up to 1 year after birth. One mutant mouse, however, suddenly showed a distinctive appearance with a hunchback posture at the age of 7 weeks (ptpup-18, 10.7% editing frequency, Fig. 2c). Unlike its siblings, this mouse did not move actively, stayed in a sitting position, and died within a week after the outbreak of this unusual phenotype. We immediately took a biopsy of kidney tissue and brown adipose tissue (BAT) to observe the morphology of the mitochondria using transmission electron microscopy. The mitochondrial structures in the kidney and BAT appeared damaged, showing imperfect cristae (Fig. 2d). Encouraged by this result, we measured oxygen consumption rates of mouse embryonic fibroblast (MEF) cells harboring the m.G12918A mutation (Additional file 1: Fig. S2). We found that oxygen consumption rates of mutant cells were decreased compared with that in the wild-type MEF cells, although not all mutant MEF cells showed a reduced oxygen consumption rate. Furthermore, among F1 and F2 mice, two mice suddenly displayed similar phenotypes (Additional file 1: Fig. S3, showing a hunchback appearance and staying in a sitting position, 2 or 8 weeks after birth (pt121 (F1) and pt221 (F2) in Additional file 1: Fig. S3, respectively, Additional file 1: Table S3) and died 1 week after showing these phenotypes. We recorded the bodyweights of hunched mice before they died (Additional file 1: Fig. S4). These mutant mice showed reduced bodyweights compared to the wild-type mice and their siblings. We also investigated whether there was a correlation between heteroplasmy levels and the hunchback phenotype (Additional file 1: Fig. S5). We did not find any statistically significant differences in m.G12918A conversion frequencies between mice without symptoms and those with the hunchback phenotype. This result indicates that the hunchback phenotype can arise with variable mutant loads, as is the case with m.G13513A in humans [21]. Furthermore, we visualized DAB-stained mouse brain sections using an anti-NeuN antibody. The m.G12918A mouse (pt121, a mutation frequency of 5.5%) showed aberrantly enlarged ventricles (Fig. 2e, top left arrow), compared to the wild-type mouse and its littermate with a mutation frequency of 4.4% (pt119 in Fig. 2e). Thus, the area ratio of lateral ventricles to the whole brain was 6.52% for pt121, whereas it was 0.5% for the wild-type mouse and 1.25% for pt119. The smaller size of the forebrain of pt121 is likely linked with the reduced bodyweight of the mouse, compared to that of the wild-type and pt119 mice. In addition, the mutant mouse showed asymmetrical hippocampal atrophy (Fig. 2e, bottom left arrow), indicative of cognitive decline, as observed in Parkinson’s disease and Alzheimer’s disease [22].

## Conclusions

In this study, we presented DdCBE-NES, which can reduce unwanted off-target editing in the nuclear DNA and increase the editing efficiency of mtDNA in mouse embryos. Co-injection of mitoTALEN further enhanced DdCBE- or DdCBE-NES-mediated mtDNA editing in mouse embryos, leading to the creation of an animal model for mitochondrial disorders with distinctive behavioral and anatomical phenotypes. Thus, our mutant mice showed damaged mitochondria in tissues and abnormal structures in the brain. We propose that these mice harboring the m.G12918A mutation, which corresponds to m.G13513A in humans, will serve as a valuable animal model for Leigh disease, MELAS syndrome, and LHON-MELAS overlap syndrome and that our approaches of using DdCBE-NES and mitoTALEN will also be useful for creating other animal models with mtDNA mutations and for the development of therapeutic agents in the future.

## Methods

### Plasmid construction

To create the plasmids encoding DdCBE-NES, we added the sequence encoding the MVM NES to the DdCBE expression plasmids from our previous study. Briefly, we amplified plasmids with mutagenesis primers for Q5 Site-Directed Mutagenesis (NEB), and the results were confirmed with Sanger sequencing. The amino acid sequence of the MVM NES is N′-VDEMTKKFGTLTIHDTEK-C′. For mitoTALEN, we used our TALEN assembly system to construct the expression vectors that lacked the NLS. We removed the fragments encoding the NLS and added fragments encoding the MTS and a 3×HA or 3×FLAG tag. DNA fragments for Gibson assembly were amplified using Q5 DNA Polymerase (NEB), gel purified, and assembled with a HiFi DNA assembly kit (NEB). For the assembly of the TALE array-encoding sequences in the DdCBE-NES and mitoTALEN plasmids, each expression plasmid was combined with appropriate module vectors (each encoding a TALE array), BsaI-HFv2 (10 U), T4 DNA ligase (200 U), and buffer in a single tube. Restriction digestion and ligation were then performed in a thermocycler, with 20 cycles of 37 °C and 50 °C for 5 min each, followed by final incubations at 50 °C for 15 min and 80 °C for 5 min. Ligated plasmids were chemically transformed into *E. coli* DH5ɑ and subjected to Sanger sequencing to analyze the identity of the constructs. Final plasmids were midi-prepped (Qiagen) for cell transfection. Primers used in this study are listed in Additional file 1: Table S4.

### mRNA preparation

The mRNA templates were prepared by PCR using Q5 High-Fidelity DNA Polymerase (NEB) with the following primers: F: 5′-CATCAATGGGCGTGGATAG-3′ and R: 5′-GACACCTACTCAGACAATGC-3′. mRNAs were synthesized using an in vitro RNA transcription kit (mMESSAGE mMACHINE T7 Ultra kit, Ambion) and purified with a MEGAclear kit (Ambion), following the manufacturer’s protocol.

### Animals

Super ovulated C57BL/6J females were mated to C57BL/6J males, and females from the ICR strain were used as foster mothers. Mice were maintained in a specific pathogen-free facility under a 12-h dark-light cycle with constant temperature (20–26 °C) and humidity (40–60%).

### Microinjection of mouse zygotes

In preparation for zygote microinjection, C57BL/6J female mice at 4 weeks of age were superovulated by intraperitoneal injections of PMSG (5 IU, Prospec) and hCG hormone (5 IU, Sigma-Aldrich) with a 48-h interval between injections. For microinjection, a mixture containing left DdCBE (or DdCBE-NES)-encoding mRNA (300 ng/μl) and right DdCBE (or DdCBE-NES)-encoding mRNA (300 ng/μl) was diluted in DEPC-treated injection buffer (0.25 mM EDTA, 10 mM Tris, pH 7.4) and injected into the cytoplasm of zygotes using a Nikon ECLIPSE Ti micromanipulator and a FemtoJet 4i microinjector (Eppendorf). For co-injection of DdCBE and mitoTALEN, we added left and right mitoTALEN-encoding mRNAs (300 ng/μl each) to the injection buffer. After injection, embryos were cultured in microdrops of KSOM+AA (Millipore) at 37 °C for 4 days in a humidified atmosphere containing 5% CO_2_. Two-cell stage embryos were implanted into the oviducts of 0.5-dpc pseudo-pregnant foster mothers.

### Genotyping

Blastocyst stage embryos and tissues were incubated in lysis buffer (25 mM NaOH, 0.2 mM EDTA, pH 10) at 95 °C for 20 min, after which the pH was adjusted to 7.4 using HEPES (free acids, without pH adjustment) at a final concentration of 50 mM. Genomic DNA was extracted from pups for PCR genotyping using a DNeasy Blood & Tissue Kit (Qiagen) and subjected to Sanger and targeted deep sequencing.

### Targeted deep sequencing

To create a high-throughput sequencing library, nested first and second PCR were performed, and final index sequences were incorporated, using Q5 DNA Polymerase. The library was subjected to paired-end read sequencing using MiniSeq (Illumina). In all cases, the paired-end sequencing results were joined into a single fastqjoin file and analyzed using CRISPR RGEN Tools (http://www.rgenome.net/).

### Whole-genome sequencing

Total genomic DNA was extracted using a DNeasy Blood & Tissue Kit (Qiagen). The library was prepped using TruSeq Nano DNA (Illumina) and sequenced using Illumina HiSeq X sequencer at Macrogen. Sequence reads were aligned by Issac Aligner, and variants were called by Issac Variant Caller. Sorted variants were filtered and counted using Microsoft Excel.

### Transmission electron microscopy

Tissues were dissected and fixed using 2.5% glutaraldehyde in 0.1 M phosphate buffer (pH 7.4) for 2 h at 4 °C followed by post-fixation using 1% osmium tetroxide for 2 h on ice [23]. Then, tissues were embedded in Epon 812 after dehydration in ethanol and propylene oxide. Polymerizations were performed using pure resin at 70 °C for 2 days; 70-nm ultrathin sections were obtained with an ultramicrotome (UltraCut-UCT, Leica), collected on 100-mesh copper grids, and stained with 2% uranyl acetate and lead citrate, accelerating voltage for transmission electron microscopy (TEM) (Technai G2 Spirit TWIN, FEI) at 120 kV. Electron micrographs were obtained at a magnification of 8000×.

### Immunohistochemistry

The mice were fixed with a perfusion of 4% paraformaldehyde (PFA). Whole brains were subsequently immersed in a 4% PFA solution for 3 days, after which they were moved to a solution of 30% sucrose in phosphate-buffered saline (PBS) for 2 days at 4 °C. To aid in visualizing brain structures, the sections were stained with antibodies recognizing the neuronal marker NeuN. The sections were blocked for 1 h at room temperature in PBS containing 3% bovine serum albumin and 0.3% Triton X-100 before incubation with a 1:1000 dilution of anti-NeuN antibody (Thermo) overnight at 4 °C. The sections were then washed in PBS and treated with streptavidin peroxidase and 3,3′-diaminobenzidine (DAB) using an IHC kit (Abcam). The sections were viewed with an Axio Scan.Z1 slide scanner (Carl Zeiss) using a 20× objective lense, 0.325 × 0.325 × 0.490 μm/pixel resolution, 24-bit pixel depth, and a scan average of 13.

### Isolation of mouse embryonic fibroblast cells

A mutant female mouse was superovulated and mated with a wild-type male C57BL/6J mouse. Then, 13–14 days after the appearance of the copulation plug, the embryos were separated from the pregnant mouse. Isolated embryos were washed with PBS, chopped into small pieces with scissors, and lysed with 0.25% trypsin-EDTA, followed by incubation at 37 °C for 15 min. Cells were moved to a 15-ml tube, mixed with culture medium (DMEM supplemented with 10% FBS), and transferred to a T25 flask. We selected and characterized adherent fibroblast cells for further analysis.

### Oxygen consumption rates

Oxygen consumption rates were measured using a Seahorse XFe96 Analyzer (Agilent) following the manufacturer’s protocol. One hundred microliters of suspended cells (10^6^ cells/ml) was seeded into Seahorse XF96 cell culture microplates (Agilent) 16 h before measurements were taken. An analysis was performed in Seahorse XF DMEM pH 7.4 supplemented with 25 mM glucose and 1 mM sodium pyruvate (Agilent). The XF cell mito stress test protocol was applied using 1.5 mM oligomycin, 0.5 mM FCCP, and 0.5 mM rotenone + antimycin A.

## Supplementary Information

Additional file 1: Table S1. Number of blastocysts used in this study. Table S2. Numbers of F_0_ m.G12918A pups obtained. Table S3. Summary of information about m.G12918A pups with a hunchback phenotype. Table S4. Primers used in this study. Figure S1. Whole-genome sequencing analysis of m.G12918A-harboring pups. Figure S2. Oxygen consumption rates of mouse embryonic fibroblast cells. Figure S3. Pups with a hunchback appearance harboring the m.G12918A mutation. Figure S4. Bodyweights of pups harboring the m.G12918A mutation. Figure S5. Analysis of the genotype of m.G12918A mutant mice with normal or hunched appearance.

Additional file 2. Review history.

## Data Availability

Supplementary information is available in the online version of the paper. The high-throughput sequencing data from this study have been deposited in the NCBI Sequence Read Archive (SRA) database under the accession code PRJNA857335 [24].
