# Supplementary material for: Enhanced mitochondrial DNA editing in mice using nuclear-exported TALE-linked deaminases and nucleases
Source: Genome Biol. 2022 Oct 12;23:211. doi: 10.1186/s13059-022-02782-z (PMC9554978; doi:10.1186/s13059-022-02782-z)
Supplement: Supplementary file 1 — Additional file 1: Table S1. Number of blastocysts used in this study. Table S2. Numbers of F0 m.G12918A pups obtained. Table S3. Summary of information about m.G12918A pups with a hunchback phenotype. Table S4. Primers used in this study. Figure S1. Whole-genome sequencing analysis of m.G12918A-harboring pups. Figure S2. Oxygen consumption rates of mouse embryonic fibroblast cells. Figure S3. Pups with a hunchback appearance harboring the m.G12918A mutation. Figure S4. Bodyweights of pups harboring the m.G12918A mutation. Figure S5. Analysis of the genotype of m.G12918A mutant mice with normal or hunched appearance. [file 13059_2022_2782_MOESM1_ESM.docx]

**Additional file 1**

**Enhanced mitochondrial DNA editing in mice using nuclear-exported TALE-linked deaminases and nucleases**

Seonghyun Lee^1,6^, Hyunji Lee^2,3,6^, Gayoung Baek^1^, Eunji Namgung^4^, Joo Min Park^4^, Sanghun Kim^2,5^, Seongho Hong^2^ and Jin-Soo Kim^1^

^1^ Center for Genome Engineering, Institute for Basic Science, Daejeon 34126, Republic of Korea.

^2^ Laboratory Animal Resource and Research Center, Korea Research Institute of Bioscience and Biotechnology, Cheongju 28116, Korea.

^3^ School of Medicine, Sungkyunkwan University, Suwon 16419, Republic of Korea.

^4^ Center for Cognition and Sociality, Institute for Basic Science, Daejeon 34126, Republic of Korea.

^5^ College of Veterinary Medicine and Research Institute of Veterinary Medicine, Chungbuk National University, Cheongju 28644, South Korea

^6^ These authors contributed equally to this work.

Correspondence should be addressed to J.-S.K. (jskim01@snu.ac.kr).

**Contents**

Table S1. Number of blastocysts used in this study.

Table S2. Numbers of F_0_ m.G12918A pups obtained.

Table S3. Summary of information about m.G12918A pups with a hunchback phenotype.

Table S4. Primers used in this study.

Figure S1. Whole-genome sequencing analysis of m.G12918A-harboring pups.

Figure S2. Oxygen consumption rates of mouse embryonic fibroblast cells.

Figure S3. Pups with a hunchback appearance harboring the m.G12918A mutation.

Figure S4. Bodyweights of pups harboring the m.G12918A mutation.

Figure S5. Analysis of the genotype of m.G12918A mutant mice with normal or hunched appearance.

**Table S1. Number of blastocysts used in this study.**

| **Treatment** | ***MT-ND5***  **(Fig. 1)** | ***MT-TrnA*** | ***MT-Rnr2*** | **m.G12918A**  **(Fig. 2)** |
| --- | --- | --- | --- | --- |
| Buffer-injected | 3 | 5 | 6 | 2 |
| DdCBE | 22 | 17 | 12 | 6 |
| DdCBE-NES | 23 | 23 | 11 | 4 |
| DdCBE+mitoTALEN |  |  |  | 6 |
| DdCBE-NES+mitoTALEN |  |  |  | 5 |

| **Treatment** | **Number of F_0_ pups** | **Number of edit positive pups** | **% edit positive** | **Editing efficency range (%)** |
| --- | --- | --- | --- | --- |
| DdCBE | 15 | 6 | 40 | 4.1~31.6 |
| DdCBE-NES + mitoTALEN | 24 | 6 | 25 | 9.26~46.2 |

**Table S2. Numbers of F_0_ m.G12918A pups obtained.**

**Table S3. Summary of information about m.G12918A pups with a hunchback phenotype.**

| **Total number of m.G1298A mutant mice** | 45 |
| --- | --- |
| **Number of mice with a hunchback phenotype** | 3 |
| **Editing efficency range in hunchback mice (%)** | 5.5 - 17.2 |
| **Age of onset of symptoms in hunchback mice (weeks)** | 2 - 8 |

**Table S4. Primers used in this study.**

| **Name** | **Sequence** | **Note** |
| --- | --- | --- |
| mtND5.G13513A-1st_F | GCCAACTAGGCCTGATAATAG | mtDNA NGS |
| mtND5.G13513A-1st_R | GGCGTTTGATTGGGTTTATG | mtDNA NGS |
| mtND5.G13513A-2nd_F | ACACTCTTTCCCTACACGACGCTCTTCCGATCTGACGAACAAGACATCCGAAA | mtDNA NGS |
| mtND5.G13513A-2nd_R | GTGACTGGAGTTCAGACGTGTGCTCTTCCGATCTGCTGTTATAGAAGTGGCGATTA | mtDNA NGS |
| nuND5.G13513A-1st_F | GTTCTTTTAGCATTTCATGTATGTACG | nuDNA NGS |
| nuND5.G13513A-1st_R | TGTTGGTTGGTGGGATGTTG | nuDNA NGS |
| nuND5.G13513A-2nd_F | ACACTCTTTCCCTACACGACGCTCTTCCGATCTGCCTGGCAGATGAACAAGAA | nuDNA NGS |
| nuND5.G13513A-2nd_R | GTGACTGGAGTTCAGACGTGTGCTCTTCCGATCTACTGTATATAGCTGTTATAGAAGTGGT | nuDNA NGS |
| mtTrnA-1st_F | ATTCCACTTCACTAACAATATTTCCAA | mtDNA NGS |
| mtTrnA-1st_R | TAGACTGTAAATCTAAACACAGAGGT | mtDNA NGS |
| mtTrnA-2nd_F | ACACTCTTTCCCTACACGACGCTCTTCCGATCTAGGACTGTAAGACTTCATCCTACA | mtDNA NGS |
| mtTrnA-2nd_R | GTGACTGGAGTTCAGACGTGTGCTCTTCCGATCTGTCTTAAGGTGATATTCATGTCGAATT | mtDNA NGS |
| nuTrnA-1st_F | AAGACTCAGAAACCCACTCT | nuDNA NGS |
| nuTrnA-1st_R | AAGCTCATATGACTGAAGTAACAG | nuDNA NGS |
| nuTrnA-2nd_F | ACACTCTTTCCCTACACGACGCTCTTCCGATCTAGGACTGTAGGACTTCATCCC | nuDNA NGS |
| nuTrnA-2nd_R | GTGACTGGAGTTCAGACGTGTGCTCTTCCGATCTGGACTCCTGAGTATGTGAAAATG | nuDNA NGS |
| mtRnr2-1st_F | CACCAATAAAGAAAGCGTTCAAG | mtDNA NGS |
| mtRnr2-1st_R | CCCAACCGAAATTTCAAACTTATAC | mtDNA NGS |
| mtRnr2-2nd_F | ACACTCTTTCCCTACACGACGCTCTTCCGATCTTTTAACGGCCGCGGTATC | mtDNA NGS |
| mtRnr2-2nd_R | GTGACTGGAGTTCAGACGTGTGCTCTTCCGATCTAATTTAAGCTCCATAGGGTCTTCT | mtDNA NGS |
| nuRnr2-1st_F | AGGACTTCTGTGCTTGGTTT | nuDNA NGS |
| nuRnr2-1st_R | CTGAAGCTGTAGAGTGCCTATG | nuDNA NGS |
| nuRnr2-2nd_F | ACACTCTTTCCCTACACGACGCTCTTCCGATCTCCCAGTGACTAAAGTTAAACTGC | nuDNA NGS |
| nuRnr2-2nd_R | GTGACTGGAGTTCAGACGTGTGCTCTTCCGATCTCTGTAGAGTGCCTATGTACAGAAA | nuDNA NGS |
| mitoTALEN_1 | GGCCCTATTCTATAGTGTCACCTAAAT | mitoTALEN Gibson cloning |
| mitoTALEN_2 | AGCATTTAGGTGACACTATAGAATAGGG | mitoTALEN Gibson cloning |
| mitoTALEN_3 | CAAGCTTGCATCTCCCTATAGTGA | mitoTALEN Gibson cloning |
| mitoTALEN_4 | TACGACTCACTATAGGGAGATGCAAGCTTGCCACCATGGCTCTTAGTCGAGCCGT | mitoTALEN Gibson cloning |
| mitoTALEN_5 | AGCATAATCAGGGACATCGTATGG | mitoTALEN Gibson cloning |
| mitoTALEN_6 | CCATACGATGTCCCTGATTATGCTGGGATCCGAATTCAAGATCTACGC | mitoTALEN Gibson cloning |
| mitoTALEN_7 | TACGACTCACTATAGGGAGATGCAAGCTTGCCACCATGGCCTCTGTCTTGACACC | mitoTALEN Gibson cloning |
| mitoTALEN_8 | TTGAATTCGGATCCCCTTATCATCATCATCCTTATAATCTATGTCGTG | mitoTALEN Gibson cloning |
| mitoTALEN_9 | GATGATGATGATAAGGGGATCCGAATTCAAGATCTACGC | mitoTALEN Gibson cloning |

**
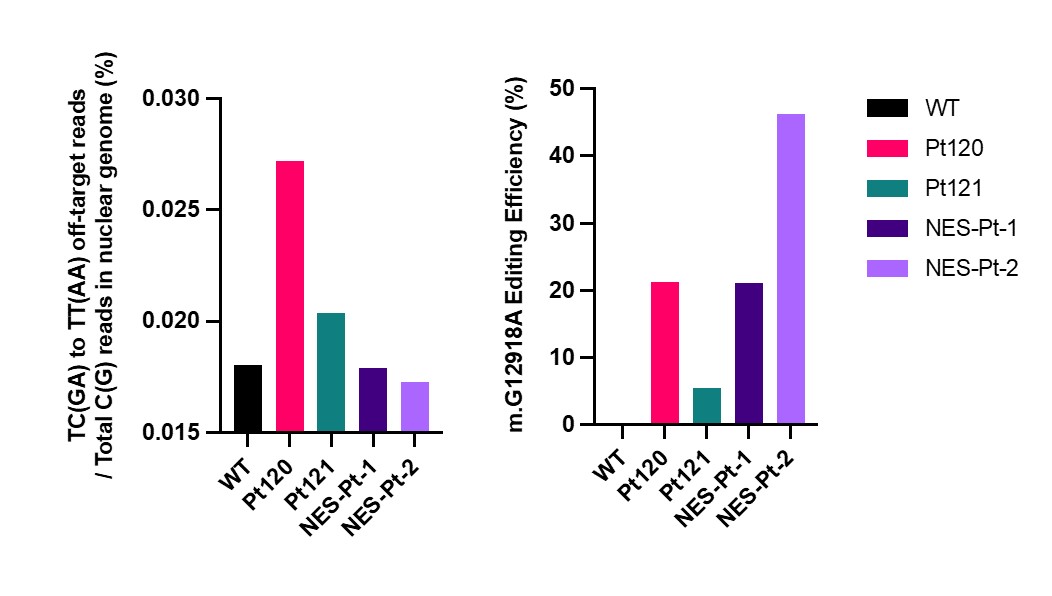
**

**Figure S1. Whole-genome sequencing analysis of m.G12918A-harboring pups.** Only 5’-TC(GA)-3’ to 5’-TT(AA)-3’ mutations in the nuclear genome were analyzed among called variants, and read depths below 20⨉ were discarded.


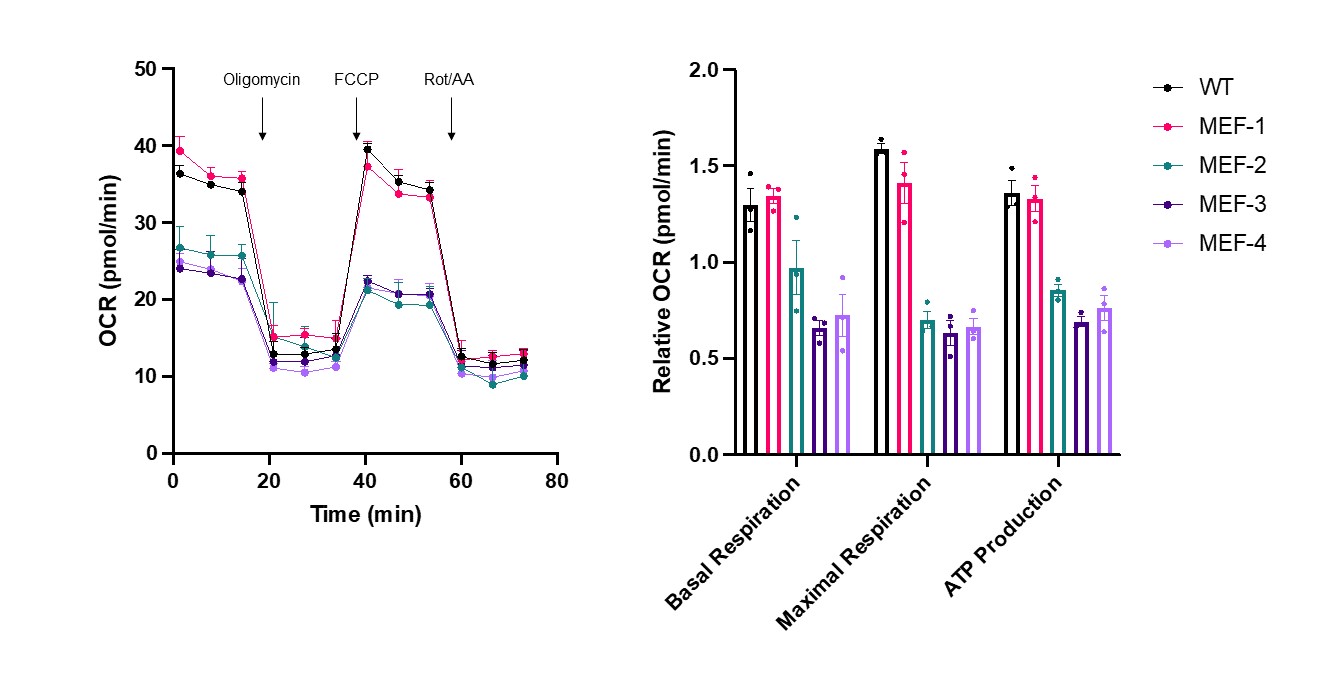


**Figure S2. Oxygen consumption rates of mouse embryonic fibroblast cells.** Each respiratory parameter was normalized to the value from the wild-type cells. Data are shown as means with standard error of the mean (SEM) from n = 5 replicates. On-target m.G12918A editing efficiencies for MEF-1, MEF-2, MEF-3, and MEF-4 are 13.2%, 10.0%, 11.6%, and 7.4%, respectively.


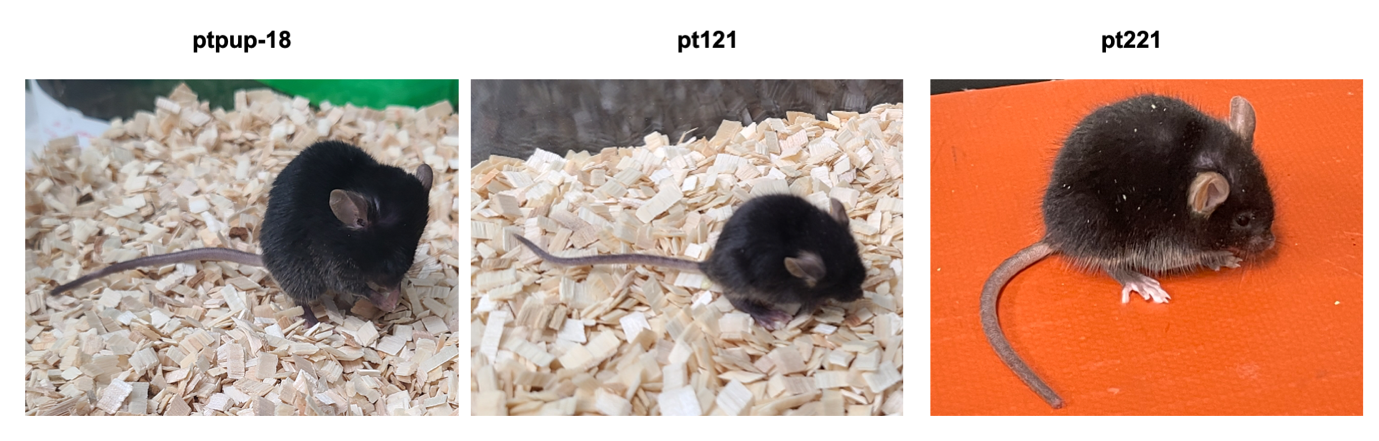


**Figure S3. Pups with a hunchback appearance harboring the m.G12918A mutation.**


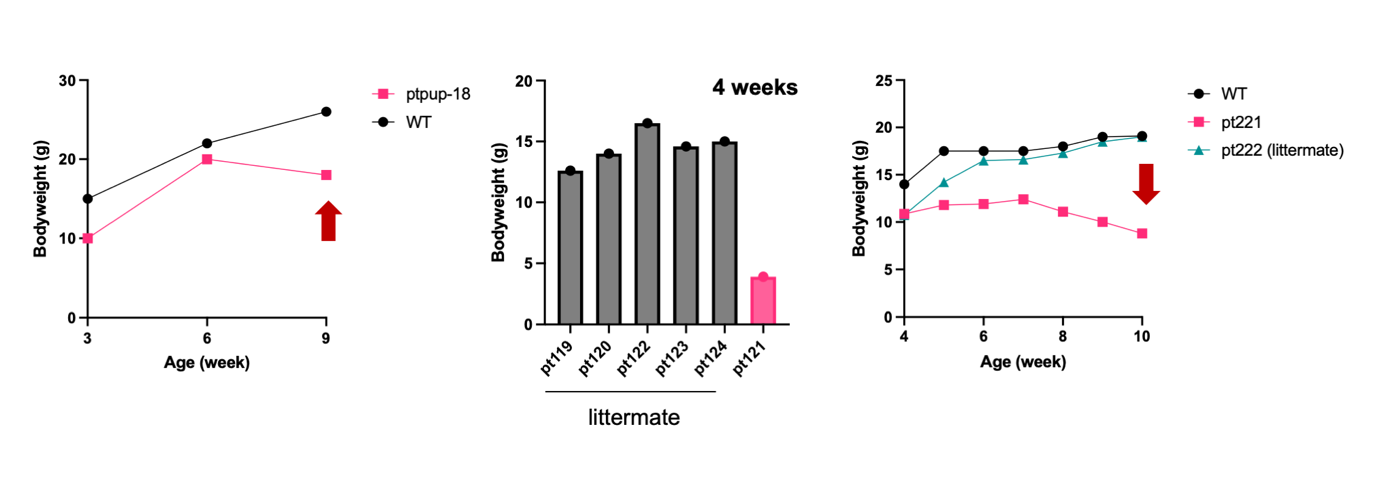


**Figure S4. Bodyweights of pups harboring the m.G12918A mutation.** Ptpup-18, pt121, and pt221 showed the hunchback appearance. Red arrows indicate the time points at which the mutant mice died.

**Figure S5. Analysis of the genotype of m.G12918A mutant mice.** The average editing frequencies were 17.8 % for mice with normal behavior, and 12.8% for mice with a hunched appearance.
