# Supplementary material for: Enhanced mitochondrial DNA editing in mice using nuclear-exported TALE-linked deaminases and nucleases
Source: Genome Biol. 2022 Oct 12;23:211. doi: 10.1186/s13059-022-02782-z (PMC9554978; doi:10.1186/s13059-022-02782-z)
Supplement: Supplementary file 2 — Additional file 2. Review history. [file 13059_2022_2782_MOESM2_ESM.docx]

Review History

**First round of review**

**Reviewer 1**

**Were you able to assess all statistics in the manuscript, including the appropriateness of statistical tests used?** Yes, and I have assessed the statistics in my report.

**Comments to author:**

In this manuscript, the authors further develop new gene editing tools for mitochondrial DNA using a mouse model system. They deploy the current dsDNA cytosine deaminase based on DddA called DdCBEs. First, they take from prior work for mitochondria nucleases and add a nuclear export signal. This does seem to increase activity in mtDNA editing. Second, they combine mitochondrial nucleases with the base editor to increase heteroplasmy in mice to demonstrate that this can be at sufficient levels to start generating new mouse models of human mitochondrial disease.

Major comments

The clever use of TALE specificity such that the mito TALENs preferentially target a differential mismatch in heteroplasmy is a nice study design.

Do the mice with phenotypes correlate with higher heteroplasmy levels than their non-phenotypic siblings? This is not clear from the presentation. This needs to be addressed to make any claims about a disease model generation step in this paper.

Phenotypic assessments beyond mitochondrial shape would need to be shown to make any real statement about a disease model.

Adding both base editors and TALENs at the same time that could potentially interact at the same locus is a complexity in this study with few controls. As running the various combinations in vivo would mean a year or more of experiments, the recommendation is to ask the authors to recognize limitations and acknowledge that they may be seeing unintentional molecular interactions.

Textual: the prior work using a nuclear export signal for mitochondrial DNA modifying proteins should be cited as precedent (i.e. Minczuk et al 2006).

**Reviewer 2**

**Were you able to assess all statistics in the manuscript, including the appropriateness of statistical tests used?** Yes, and I have assessed the statistics in my report.

**Comments to author:**

In this manuscript, Lee et al. developed improved DddA-derived cytosine base editors (DdCBEs) by fusing DdCBEs to a nuclear export signal (DdCBE-NES) and co-injected mitochondrial genome targeted TALENs (mitoTALENs) in mouse embryos to reduce off-target (OT) C-to-T conversions in the nuclear genome and improve editing efficiency in mtDNA. The authors also used the above methods to create a stable mouse mitochondrial disease model. As DdCBEs has a great potential for treating mitochondrial genetic disorders in the future, this study is timely and important in the field of mtDNA editing and therapy. I support the publication of this study after the following concerns are addressed.
1. The authors compared the editing efficiencies and OT mutations induced by DdCBE and DdCBE-NES targeting TrnA and Rnr2. Though DdCBE-NES induced higher editing efficiencies than DdCBE, no OT mutations were induced by these two editors. Thus, the authors are suggested to compare the OT mutation induced by DdCBE and DdCBE-NES at other OT sites, e.g., some reported OT sites for DdCBE.
2. Fig. 1d, one data point shows that the DdCBE-NES targeting TrnA induced ~5% mutation frequency at a nuclear DNA OT site. The authors are suggested to explain this.
3. Recent studies showed that DdCBEs can also induce TAS-independent OT mutations. The authors are suggested to determine whether DdCBE-NES can reduce TAS-independent OT mutations.
4. Fig. 2e, the forebrain of pt121 looks significantly smaller than those of WT and pt119. Is it a pathological phenotype induced by the mtDNA mutation? The authors are suggested to explain this phenotype with more details.
5. Supplementary Figure S1, the hunchback behavioral phenotypes of offspring pt121 and pt221, especially pt221, are not as obvious as that of ptpup-18. The authors are suggested to further determine other pathological phenotypes of pt121 and pt221 to confirm that the m.G12918A mutations indeed constructed the disease model in these two offspring mice.

**Authors Response**

**Point-by-point response**

**We would like to thank the reviewers for their helpful comments. To address the issues raised by the reviewers, we have now included new experimental data. Added or edited sentences are underlined in the main text for clarity.**

*Reviewer #1:
Do the mice with phenotypes correlate with higher heteroplasmy levels than their non-phenotypic siblings? This is not clear from the presentation. This needs to be addressed to make any claims about a disease model generation step in this paper.*

**Response: We compared heteroplasmy levels for mice with and without the hunched phenotype, but there was no statistical correlation between the two groups. We have now added these data in Fig S5, and the following explanation on p. 7: “We also investigated whether there was a correlation between heteroplasmy levels and the hunchback phenotype (Fig. S5). We did not find any statistically significant differences in m.G12918A conversion frequencies between mice without symptoms and those with the hunchback phenotype. This result indicates that the hunchback phenotype can arise with variable mutant loads, as is the case with m.G13513A in humans [21].”**

*Phenotypic assessments beyond mitochondrial shape would need to be shown to make any real statement about a disease model.*

**Response: Please note that we showed brain anatomy of pt121 as described in Fig. 2. In addition, we recorded the bodyweight of hunchback behavioral mice before they died, and measured oxygen consumption rates using embryonic fibroblast cells. We have now added the data in the Supplementary Figures and described these results in the manuscript as follows:**

**On page 6, “Encouraged by this result, we measured oxygen consumption rates of mouse embryonic fibroblast (MEF) cells harboring the m.G12918A mutation (Fig. S2). We found that oxygen consumption rates of mutant cells were decreased compared with that in the wild-type MEF cells, although not all mutant MEF cells showed a reduced oxygen consumption rate.”**

**On page 7, “We recorded the bodyweights of hunched mice before they died (Fig. S4). These mutant mice showed reduced bodyweights compared to the wild-type mice and their siblings.”**

**Fig. S2,**

**Fig. S4,**

*Adding both base editors and TALENs at the same time that could potentially interact at the same locus is a complexity in this study with few controls. As running the various combinations in vivo would mean a year or more of experiments, the recommendation is to ask the authors to recognize limitations and acknowledge that they may be seeing unintentional molecular interactions.*

**Response: We appreciate this reviewer’s suggestion, so we have added an explanation in the main text on p. 5: “Due to adjacent DNA binding sites, it is possible that mitoTALENs interfere with DdCBEs. Nevertheless, our results showed that simultaneous use of DdCBEs and mitoTALENs could enhance the mutation frequency in mouse embryos.”**

Textual: the prior work using a nuclear export signal for mitochondrial DNA modifying proteins should be cited as precedent (i.e. Minczuk et al 2006).
**Response: We now have added the missing reference on p. 3: “…To this end, we attached the NES sequence, N’- VDEMTKKFGTLTIHDTEK -C’, derived from the NS2 protein of Minute virus of mice (MVM NES)[17]”**

**17.	Minczuk M, Papworth MA, Kolasinska P, Murphy MP, Klug A: Sequence-specific modification of mitochondrial DNA using a chimeric zinc finger methylase. *Proceedings of the National Academy of Sciences* 2006, 103:19689-19694.**

Reviewer #2:

1. The authors compared the editing efficiencies and OT mutations induced by DdCBE and DdCBE-NES targeting TrnA and Rnr2. Though DdCBE-NES induced higher editing efficiencies than DdCBE, no OT mutations were induced by these two editors. Thus, the authors are suggested to compare the OT mutation induced by DdCBE and DdCBE-NES at other OT sites, e.g., some reported OT sites for DdCBE.

**Response: We analyzed three TALE-dependent OT sites for each of three genes (*MT-ND5*, *MT-TrnA*, and *MT-Rnr2*) using targeted deep sequencing, but unfortunately, we failed to detect off-target mutations at these sites. Instead, we have conducted whole-genome sequencing of DNA from adult mice derived from the DdCBE or DdCBE-NES microinjected embryos. These results revealed that DdCBE-treated mice showed elevated off-target editing rates compared to the wild-type. However, despite a higher on-target editing efficiency, DdCBE-NES treated mice have off-target rates that are comparable with those in the wild-type mouse. Please note that the off-target editing rates seem related to the on-target editing frequencies in DdCBE treated mice but not in DdCBE-NES treated mice. Further, a recent study (Lei et al. Nature 2022) also demonstrated that DdCBE-NES could reduce both TAS-independent and TAS-dependent off-target mutations. We have added these data to the manuscript as shown below:**

**On page 4, “Furthermore, we conducted whole-genome sequencing for mice injected with DdCBE or DdCBE-NES. A recent study revealed that the use of NES largely avoided TALE-dependent and TALE-independent off-target editing in human cells [18]. Therefore, we chose only DdCBE-mediated mutations in nuclear DNA, 5’-TC(GA)-3’ to 5’-TT(AA)-3’, then filtered out homozygous mutations and read depths below 20×. The off-target read ratio in the nuclear genome was 0.018% for the wild-type C57BL/6J mouse. However, DdCBE-treated mice showed elevated levels of off-target mutations, such that the off-target ratios were 0.027% and 0.020% for Pt120 and Pt121, respectively (Fig. S1). On the other hand, mice microinjected with mRNA encoding DdCBE-NES (NES-Pt) demonstrated comparable off-target ratios with the wild-type, such that the ratios were 0.018% and 0.017% for NES-Pt-1 and NES-Pt-2, respectively. Note that NES-Pt mice showed higher on-target m.G12918A editing efficiencies than DdCBE-treated mice, but the off-target ratios were on par with those in the wild-type.”**

2. Fig. 1d, one data point shows that the DdCBE-NES targeting TrnA induced ~5% mutation frequency at a nuclear DNA OT site. The authors are suggested to explain this.

**Response: Indeed, nuclear off-target editing may or may not occur depending on individual embryos. As indicated in the figure, we did not find any statistically significant differences between treated vs. mock-treated embryos.**

3. Recent studies showed that DdCBEs can also induce TAS-independent OT mutations. The authors are suggested to determine whether DdCBE-NES can reduce TAS-independent OT mutations.

**Response: We agree that Detect-seq and ATAC-seq are powerful methods. Please note that it could take several months to set up these methods, which, we believe, is beyond the scope of our current study. Furthermore, Lei et al. have already showed that DdCBEs fused to NES can reduce or avoid TAS-independent mutations. As described above, we have conducted whole-genome sequencing to assess off-target activities of DdCBE and DdCBE-NES.**

4. Fig. 2e, the forebrain of pt121 looks significantly smaller than those of WT and pt119. Is it a pathological phenotype induced by the mtDNA mutation? The authors are suggested to explain this phenotype with more details.

**Response: The smaller size of the forebrain of pt121 is likely linked with the reduced bodyweight of the mouse, compared to that of the wild-type and pt119 mice. We have now added the data to the manuscript and mentioned these points on p. 6-7 as follows: “We recorded the bodyweights of hunched mice before they died (Fig. S4). These mutant mice showed reduced bodyweights compared to the wild-type mice and their siblings.”**

5. Supplementary Figure S1, the hunchback behavioral phenotypes of offspring pt121 and pt221, especially pt221, are not as obvious as that of ptpup-18. The authors are suggested to further determine other pathological phenotypes of pt121 and pt221 to confirm that the m.G12918A mutations indeed constructed the disease model in these two offspring mice.

**Response: We have now changed the images for pt121 and pt221 for clarity (now Fig. S3). Also, we have added oxygen consumption rates and the bodyweights of mice exhibiting the hunchback behavior in Figs. S2 and S4. Unfortunately, these three mice have already died, so we could not proceed with further analysis. Please note that the hunchback behavior appears suddenly, and such mice die shortly thereafter.**

**Fig. S2,**

**Fig. S3,**

**Fig. S4,**

**Second round of review**

**Reviewer 1**

The revisions have addressed all major concerns.

**Reviewer 2**

This manuscript has been well revised and my previous questions has been fully addressed. I recommend to publish it.
